# Anodyne Cement for Rendering Teeth Insensible to Pain

**Published:** 1855-02

**Authors:** 


Anodyne Cement for rendering Teeth insensible to Pain.—Mr. J. P. Clark, of London, with the object of rendering diseased teeth insensible to pain, previous to the operation of stopping them permanently with metal, or filing away the carious parts, recommends the application of a paste, composed of Canada balsam and slaked lime, which is to be inserted into the hollow tooth like a pill. Mr. Clark states, that this preparation affords immediate relief in all but chronic cases of inflammation. He says:—

"If fresh pills be inserted as often as the old ones wear out, or are removed on the return of pain, in order, like an abscess, to allow the escape of matter or blood, and this practice be continued the teeth will become as insensible to touch as the soundest, and may then be permanently stopped, and otherwise treated in the usual way, without the infliction of pain."—*Dublin Hospital Gazette.*

